# Home care for the elderly with dementia: a systematic review

**DOI:** 10.1590/1980-5764-DN-2022-0052

**Published:** 2023-11-10

**Authors:** Luísa Pelucio, Marcia Cristina Nascimento Dourado, Laiana Azevedo Quagliato, Antonio Egidio Nardi

**Affiliations:** 1 Universidade Federal do Rio de Janeiro, Instituto de Psiquiatria, Depression Resistant Ambulatory, Rio de Janeiro RJ, Brazil.; 2 Universidade Federal do Rio de Janeiro, Instituto de Psiquiatria, Rio de Janeiro RJ, Brazil.

**Keywords:** Home Care Services, Aged, Disability Evaluation, Dementia, Rehabilitation, Serviços de Assistência Domiciliar, Idoso, Avaliação da Deficiência, Demência, Reabilitação

## Abstract

**Objetive:**

With the global population aging, there is a growing need for home-based care to meet the health needs of the elderly. However, the quality of care provided to the aged population is now arguably a significant challenge for most healthcare systems worldwide.

**Methods:**

The present review included 13 original studies on home care and its effects on dementia patients, describing how patient care and adequate treatment can be collaborative for their improvement, for case management, and optimizing pain control and specificities.

**Results:**

Among the findings, it was evidenced that the environment impacts the form of care, once being at home can improve communication and global monitoring of dementia patients.

**Conclusion:**

In addition to the results analyzed in this review, there is a need for future, well-designed studies on the different aspects of home care, highlighting the importance of evaluating the type of care for each patient in the quest to optimize the care.

## RESUMO

**Objetivo:**

Com o envelhecimento da população mundial, há uma necessidade crescente de cuidados domiciliares para atender às necessidades de saúde dos idosos. No entanto, a qualidade dos cuidados prestados à população idosa é hoje indiscutivelmente um grande desafio para a maioria dos sistemas de saúde em todo o mundo.

**Métodos:**

A presente revisão incluiu 13 estudos originais sobre cuidados domiciliares e seus efeitos em pacientes com demência, descrevendo como o cuidado e o tratamento adequado do paciente podem ser colaborativos para sua melhora, para o gerenciamento de casos e a otimização do controle de sua dor e especificidades.

**Resultados:**

Entre os achados, evidenciou-se que o ambiente impacta a forma de atendimento, pois estar em casa pode melhorar a comunicação e o acompanhamento global dos pacientes em quadro demencial.

**Conclusão:**

Além dos resultados analisados nesta revisão, há a necessidade de futuros estudos bem elaborados sobre os diferentes aspectos da atenção domiciliar, destacando a importância de avaliar o tipo de cuidado de cada paciente na busca de otimizar o cuidado.

## INTRODUCTION

As the global population ages, there is a growing need for home-based care to address the health needs of older adults, once with the aging, it increases the number of patients with adverse symptoms requiring care. According to the United States Census data, one in five Americans will be older than 65 years by 2050^1^. As reported by some authors^2^, there were already 14 million people over 65 with some kind of disability in the year 2000, with only slightly more than 10% of them residing in nursing homes. These statistics show an increased need for home care in the coming years^3^.

Home care is designed to enable older adults to remain safely in their own homes for as long as possible by providing services such as nursing, rehabilitation, and personal support such as bathing, transferring, repositioning and grooming assistance, as well as the caregiver respite and support^3,4^.

Therefore, the quality of care provided to the aged population today is possibly a major challenge for most healthcare systems worldwide^5^.

Home-based health care can reduce hospital costs and humanize health practices^6,7^. Chronic, non-communicable diseases associated with population aging may cause limitations with temporary or permanent functional disabilities^8^. It is thus a challenge for health managers and society to find alternative care strategies to meet the specific demands of the elderly and their families^9,10^.

Despite discussions and formulation of specific policies for the elderly, changes remain incipient^11^. The release of guidelines for primary care of the elderly is an attempt to translate these theoretical discussions into health practices^8^. Although some qualitative studies have explored home health care and hospital care at home, gaps remain in the factors associated with care of the elderly at home, access to care, and involvement of health providers in these care services^12^.

The increased likelihood of being admitted to a care home facility following an acute hospital stay is considered indicative of poor-quality care and should be reduced by a period of care at home. Older age, female gender, vascular dementia, incontinence, fall, hip fracture, and a number of comorbidities are among the risk factors for long-term residential care. Therefore, the dementia review may help to reduce the risk of long-term care home placement following acute hospital admission^13^.

The aim of this study was to carry out a systematic review of patients with dementia treated at home. The global population aging causes a growing need for home care, which makes the quality of care provided to the elderly population a major challenge for most health systems in the world today. Therefore, it is necessary to evaluate the effectiveness of home care in elderly patients diagnosed with dementia.

## METHODS

This systematic review was performed according to the Preferred Reporting Items for Systematic Reviews and Meta-Analyses (PRISMA). A systematic search was conducted in the MEDLINE (PubMed), Scielo, and Elsevier databases on articles presenting studies on home care, published between January 1, 2011, and November 19, 2021, in English to reduce the effect of using only one language in the search. The following keywords were used: “home care”, “elderly”, “disability”, “dementia”, and “rehabilitation”, with the applications “home care” and “elderly” and “rehabilitation”, “home care” and “senior” and “dementia” and “home care” and “elderly” and “disability”. Published systematic reviews and meta-analyses were screened for additional studies.

Studies were selected for data extraction and analysis based on the following inclusion criteria:

- Original research studies on humans and home care;
- Subjects met dementia criteria based on a conventional psychiatric classification system;
- Patients with comorbidities, only with dementia as priority.

The articles included in the analysis were randomized trials, randomized controlled trials, cross-sectional trials, clinical trials, single-arm clinical trials, case reports, observational studies, and retrospective cohort studies all focused on home care with dementia, disability, rehabilitation and caregivers.

The following exclusion criteria were used in the search:

- Participants with comorbidity or additional psychiatric diagnosis that would confound with dementia;
- Studies that lacked home care in the treatment;
- Human studies with participants under 60 years old;
- Studies developed in hospitals or institutions;
- Articles that focused on home care for different diseases of patients at home;
- Programs that only seek to reduce costs of patients at home; and
- Articles that did not aim to assess home care or clinical aspects of patients with dementia, disability, or rehabilitation.

Therefore, studies that included elderly patients with depression, brain stroke or epilepsy were excluded because they are common confounders of dementias.

Two authors selected the included studies (LP and LAQ) and the other two solved the discrepancies (MCND and AEN).

The following variables were extracted from all the studies: authors, year of publication, study design, and main outcome. For quality assessment it was used the Newcastle-Ottawa Scale. LP and LAQ extracted the data, and all authors agreed with the final inclusion of studies in the systematic review, data abstraction, and quality assessment.

The 13 selected articles included: two randomized trials, three cross-sectional trials, one case report, five clinical trials, one retrospective cohort study, and one observational sectional study. See Tables 1 and 2 for the list of included articles^13-25^.

**Table 1.** Description and organization of articles.

Author	Design	Sample	Objective	Main results
Bonnefoy et al.,^15^	Randomized trial	102 participants	To assess whether a home-based program supervised by home helpers during their normal working hours can prevent excessive sedentariness and preserve functional status in elderly people at risk of frailty or disability and using domestic services.	This study confirmed the feasibility of a supervised prevention program and some benefit from the intervention and identifies predictors for better compliance
Darton et al.,^19^	Cross-sectional	Residents of 19 extra care schemes with a comparative sample of people admitted to care homes.	This paper presents findings on the characteristics of the residents at the time of moving in, drawing on information collected from the 19 schemes in the evaluation, and a recent comparable study of residents who moved into care homes providing personal care.	Although extra-care housing may be operating as an alternative to care homes for some individuals, it is providing for a wider population, who may be making a planned move rather than reacting to a crisis.
Fleming et al.,^23^	Case report	1 patient	To understand the fragmentation of care, characterized by miscommunications and lack of follow-up, which can lead to oversights in diagnosis and management.	Reduced hospital readmissions and a critical role for the home health industry in improving patient outcomes and reducing costs.
Wu et al.,^14^	Cross-sectional	2,608 participants	To investigate determinants of long-term care use and to clarify the differing characteristics of home/community-based and institution-based services users.	Age, single marital status, stroke, dementia, and ADL disability are predictive factors for long-term care use. The utilization was directly proportional to the level of disability.
Dorresteijn et al.,^16^	Randomized controlled trial	389 people	To assess the effectiveness of a home-based cognitive behavioral program on concerns over falls, in frail, older people living in the community.	At 12 months, the intervention group showed significantly lower levels of concerns over falls compared to the control group. Furthermore, significant reductions in activity avoidance, disability and indoor falls were identified in the intervention group compared with the control group.
Kamitani et al.,^21^	Observational study	40 patients	To develop and validate a scale that assesses quality of life in patients receiving home-based medical care.	The scale’s internal consistency was confirmed, as was its external validity.
Kasteridis et al.,^13^	Cross-sectional	170,387 patients	To run multi-level logit models to assess the impact of the Quality and Outcomes Framework, or QOF review, on the risk of care home placement following emergency admission to hospital.	The QOF dementia review may help to reduce the risk of long-term care home placement following acute hospital admission.
Szanton et al.,^17^	Single-arm clinical trial	2,217 participants	To determine whether the Community Aging in Place, Advancing Better Living for Elders (CAPABLE) program saves Medicaid more money than it costs to provide.	CAPABLE is associated with lower likelihood of inpatient and long-term service use and lower overall Medicaid spending. The magnitude of reduced Medicaid spending could pay for CAPABLE delivery and provide further Medicaid program savings due to averted services use.
Watanabe et al.,^18^	Clinical trial	178 patients	To investigate the medical health of older adults receiving home medical care services.	Dysphagia risk predicts the first unexpected hospitalization in older individuals receiving home medical care.
Kim et al.,^25^	Retrospective cohort study	7,112 people	To investigate the association between the type of long-term care service and the incidence of hip fracture among older adults with dementia receiving long-term care insurance, and to investigate how said association differs according to characteristics of beneficiaries and structural characteristic of institutional care.	Institutional care was more likely to be associated with a higher incidence of hip fracture than home care.
Husebo et al.,^22^	Clinical trial	315 participants	The article describes the rationale, development, feasibility testing, and implementation process of the LIVE@Home.Path trial.	As the article concludes, we hope that implementing LIVE will lead to a pathway to dementia treatment and care that is cost-effective, viable, and supports independent living at home. Does not demonstrate other results.
Barker et al.,^24^	Clinical trial	621 patients	To compare the outcomes from a traditional outpatient physiotherapy model with those from a home-based rehabilitation program for people assessed as being at risk of a poor outcome after knee arthroplasty.	Found no important differences in outcomes when post-arthroplasty rehabilitation was delivered using home-based rehabilitation. However, the health economic analysis found that when adopting a societal perspective, home-based rehabilitation had a 75% probability of being cost-effective at a threshold of £30,000 per quality-adjusted life-year
Vislapuu et al.,^20^	Clinical trial	105 patients	To investigate the consequences of restrictions on formal (homecare staff) and informal (family, friends) resource utilization among co-residing (e.g., spouses) and visiting caregivers (e.g., children).	The care situation changed dramatically in the early phase of the COVID-19 pandemic, especially for those living alone who received less support from homecare services and visiting caregivers.

**Table 2.** Quality analysis.

Quality criteria	Autor	Selection (****)	Comparability (*)	Outcome(*)
Selection/design				
Randomized trial	Bonnefoy et al.,^15^	***	*	*
Retrospective cohort study	Kim et al.,^25^	****	*	*
Cross-sectional	Darton et al.,^19^	**	*	*
Cross-sectional	Wu et al.,^14^	****	*	*
Cross-sectional	Kasteridis et al.,^13^	***	*	*
Case report	Fleming et al.,^23^	**	*	*
Randomized controlled trial	Dorresteijn et al.,^16^	**	*	*
Observational study	Kamitani et al.,^21^	***	*	*
Single-arm clinical trial	Szanton et al.,^17^	****	*	*
Clinical trial	Watanabe et al.,^18^	**	*	*
Clinical trial	Husebo et al.,^22^	**	*	*
Clinical trial	Barker et al.,^24^	***	*	*
Clinical trial	Vislapuu et al.,^20^	**	*	*

Note: *is the quality variable.

## RESULTS

**Figure 1.**
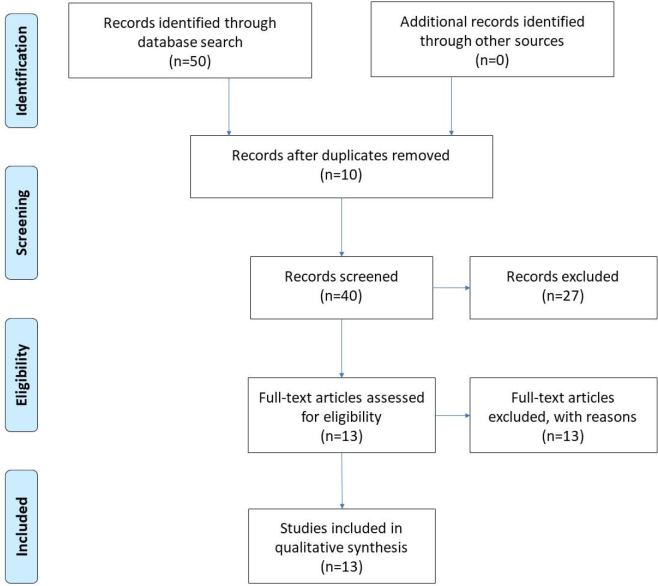
PRISMA 2009 flow diagram.

The use of PRISMA guidelines and a systematic search of electronic databases yielded a total of 50 studies. No additional studies were identified through a manual search of references. After the elimination of duplicates, 40 articles were reviewed, 27 of which were excluded for being outside the proposed theme or subject, remaining 13 selected articles for review (Figure 1).

Within this review, the research and studies of the articles that were found described how patient care and adequate treatment of the patient can be collaborative for their improvement, case management, and optimizing pain control and specificities.

One study investigated the determinants of long-term care use to clarify the differing characteristics of home/community-based and institution-based service users in a national sample of 2,608 people aged 65 years and older. This study showed that age, single marital status, stroke, dementia, and activities of daily living (ADL) disability were predictive factors for long-term care use, which were directly proportional to the level of disability^17^.

These cited articles and others show how home care programs are favorable to health and that improve the patient’s quality of life. From a different perspective, care is to pledge time to patient improvement in home care.

A recent study examined data obtained in the Observational Study of Nagoya Elderly with Home Medical Care (ONEHOME), which investigated the health of older adults receiving home medical care services in Nagoya City, in Japan. The data analyzed were patients’ age, sex, number of medications, and results from the Dysphagia Severity Scale, Charlson Comorbidity Index, Barthel Index, Mini Nutritional Assessment — Short Form, Frailty Index, and dementia independent living scale. Cox regression analysis with adjustment for age, sex, Charlson Comorbidity Index, Barthel Index, and Mini Nutritional Assessment — Short Form scores showed that a lower Dysphagia Severity Scale score was significantly associated with unexpected hospitalization. This reinforces that home care is a factor in the preservation and clinical improvement of the patient involved^21^. Another study shows that hip fracture among older adults is a major health issue, but it can be preventable if provided with proper care. It is a cohort data set of Korean senior citizens collected between 2008 and 2013 from 7,112 long-term care insurance beneficiaries with dementia aged 60 years or over. It was found that home care was less likely to be associated with hip fracture and that institutional services should be monitored for quality and improvements^22^.

In another study, a home-based program supervised by home helpers during their normal working hours was evaluated regarding the prevention of excessive sedentariness (mainly maximum walking time and distance) and the preservation of functional status in elderly people using domestic services who were susceptible of frailty or disability. The study confirmed the feasibility of prevention and some benefit from the intervention, identifying predictors for better compliance, which will help in the design of prevention trials for the elderly at risk of frailty^14^.

Concerns over falls are common among the elderly^18^. These concerns, also referred to as fear of falling, can have serious physical and psychosocial consequences, such as functional decline, increased risk of falls, activity restriction, and lower social participation. A study showed the effectiveness of a home-based cognitive behavioral program on concerns over falls in frail older people living in the community. In a randomized controlled trial in the Netherlands, 389 people aged 70 years and older, in fair or poor perceived health, who reported at least some concerns over falls and related activity avoidance were allocated to a control (n=195) or intervention group (n=194). The mentioned program significantly reduced the concerns over falls, related activity avoidance, disability, and indoor falls in those people. This program may prolong independent living and provide an alternative for those who are unable or unwilling to attend group programs^18^.

There is little information available regarding cost reductions on programs that aim to decrease the impact of disability in older adults. However, a study on the Community Aging in Place — Advancing Better Living for Elders (CAPABLE) program analyzed whether the savings to Medicaid were greater than the costs involved in providing the program. In addition, another study examined data obtained in the ONEHOME, which investigated the medical health of older adults receiving home medical care services in Nagoya City, Japan, also found a decrease in the costs as well as in contamination risks in the online service format^20,21^.

While institutional care provides support to residents with cognitive functioning problems, most schemes appear to prefer them to move in when they can still become familiar with their new accommodation before the development of more severe cognitive impairment^15^.

Another article investigated the consequences of the COVID-19 lockdown for home care – regarding formal (homecare staff) and informal (family, friends) resource utilization – among co-residing (e.g., spouses) and visiting caregivers (e.g., children). The care situation for people with dementia changed dramatically in the early phase of the COVID-19 pandemic, especially for those living alone who received less support from homecare services and visiting caregivers. For future crises and a forthcoming post-pandemic period, health authorities must plan better, identify and prioritize those in greatest need^25^.

There is still much to be researched and investigated regarding care at home and its changes, whether in relation to the pandemic, hospitalizations, readmissions, or extra institutions/housing. As mentioned above, these patients need active care, primarily to ensure that these changes do not adversely affect their health or clinical condition.

In this review, articles were also found that used testing as an objective that may assist in the treatment and management of patients in home care.

A related article validated a scale to assess the quality of life of patients in home medical care, confirming internal consistency and external validation. In another article, the impact of the quality structure on the results of patients with dementia was evaluated, and a final study, which used a LIVE@Home. Path test, was expected to lead to a pathway to dementia care and treatment that is cost-effective, viable, and supportive of independent living at home^19,23^.

Table 1, shows the description and organization of each article, which included: author, year, design sample, main objective and result. This form has a summary of all the research and a familiarity with the findings in a systematic and grouped way.

## DISCUSSION

The aim of this systematic review was to understand patients with dementia being treated at home. Based on the 13 articles reviewed, several important findings emerged.

Among the studies found and analyzed, it was perceived that some articles reported the importance of considering the patient’s environment, demonstrating several differences and characteristics of care and treatment depending on whether the patient was hospitalized, at home, or in an institution. These studies demonstrated different impacts on the lives of the elderly and even on their communication and global monitoring^14,15^.

Regarding the findings, researches described the characteristics of patient care and treatment formats, such as care supervision, informal and family care, subjective differences, quality of life of patients at home, and rehabilitation treatments^12,13,16,19,21,24,25^.

There were also articles that cited the costs and benefits of home treatment, covering topics such as how to avoid hospital readmissions; programs to increase the treatment of the elderly at home; reduction or increase in care regarding falls; assessing the elderly health before the first hospitalization; and evaluating the minimization of home-treatment costs compared to hospital. These studies demonstrated the importance of evaluating the type of treatment for each patient and seeking to optimize this type of care^13,16,18-21,23,25^.

Discussing and detailing these studies are very important for the literature, as it may promote further studies and provide space to talk about the elderly and home care. It can be observed that with the increased life expectancy, the population that most grows today is that of the elderly, with people living longer and requiring greater care with more specific and humanized treatments.

Home care is a treatment that helps minimize financial costs and holds the confidence that the patients can be close to the family at home, allowing them to feel more comfortable and calmer to face the necessary treatments. Hence, the increasing demand for home care, whereby people can be treated and cared for at home with increasingly good resources and programs to improve such services.

It is necessary to continue giving space to this theme and to this population, since the growing increase in the urgency for this kind of care can be seen in our current reality.

New practices are needed that can sustain the increased demand for home care described above. There is, consequently, a need for new research, testing, increased networks of care by professionals, or even the promotion of informal means, such as family members and a community support network. It should be understood that care research needs to have a genuine impact by leading to care from more prepared professionals and greater solidity in home treatment. The limitation of this review is the heterogeneity of methodology, results and findings in the research bases.

In this review, the findings may help us to understand the prevalence of the dementia population, or as mentioned in some articles, a population with cognitive impairment that needs long-term care and who has been directed to their home nowadays, what makes us highlight and still desire for research in this care format, understanding the prevalence and possible scientific advances^13,15,16^.

In conclusion, there is a great and growing need for the care of elderly patients at home. The studies eligible for this review demonstrated, from different perspectives, that home care for the elderly with dementia can help avoid readmissions to hospital, reduce costs, bring benefits to their current and family life, in addition to optimizing their life and health.

It is essential that more articles are developed to deepen research in the field and bring greater understanding of home care and the possible perspectives and characteristics in caring for the elderly with dementia. Only then will the patient’s needs and the required care be understood, in regard to which professionals and functions can contribute to the maintenance, optimization, and quality of life of the patient.
